# Sequentially appearing erythema nodosum, erythema multiforme and Henoch-Schönlein purpura in a patient with *Mycoplasma pneumoniae* infection: a case report

**DOI:** 10.1186/1752-1947-6-398

**Published:** 2012-11-23

**Authors:** Masaki Shimizu, Yasuhito Hamaguchi, Takashi Matsushita, Yasuhisa Sakakibara, Akihiro Yachie

**Affiliations:** 1 Department of Pediatrics, School of Medicine, Institute of Medical, Pharmaceutical, and Health Sciences, Kanazawa University, 13-1 Takaramachi, Kanazawa, 920-8641, Japan; 2 Department of Dermatology, School of Medicine, Institute of Medical, Pharmaceutical, and Health Sciences, Kanazawa University, 13-1 Takaramachi, Kanazawa, 920-8641, Japan

**Keywords:** Mycoplasma pneumonia, Erythema nodosum, Erythema multiforme, Henoch-Schönlein purpura

## Abstract

**Introduction:**

A wide variety of skin manifestations are associated with *Mycoplasma pneumoniae* infection. However, the precise mechanisms by which *M. pneumoniae* infection is able to produce a variety of cutaneous manifestations are poorly understood.

**Case presentation:**

An 8-year-old Japanese girl presented with sequential skin manifestations, including erythema nodosum, erythema multiforme and Henoch-Schönlein purpura. Although a chest radiograph showed no significant lung abnormalities, serological examinations revealed that these skin manifestations were associated with *M. pneumoniae* infection.

**Conclusion:**

It has been reported that the variations in cutaneous manifestations of *M. pneumoniae* infection can be attributed to the immaturity of the adaptive immunity of a host. However, the case presented herein indicates that skin manifestations might not be specific for each individual. An awareness of the varied patterns of cutaneous disease is essential for the early diagnosis and treatment of patients with manifestations of *M. pneumoniae* infection.

## Introduction

A wide variety of skin manifestations are associated with *Mycoplasma pneumoniae* infection [1]. It has been reported that the variations in cutaneous manifestations of *M. pneumoniae* infections can be attributed to the immaturity of the adaptive immunity of a host [2]. However, the precise mechanisms by which *M. pneumoniae* infection is able to produce a variety of cutaneous manifestations are poorly understood. In this report, we describe the case of a patient with sequentially appearing skin manifestations, including erythema nodosum, erythema multiforme and Henoch-Schönlein purpura, associated with *M. pneumoniae* infection.

## Case presentation

**Figure 1:**
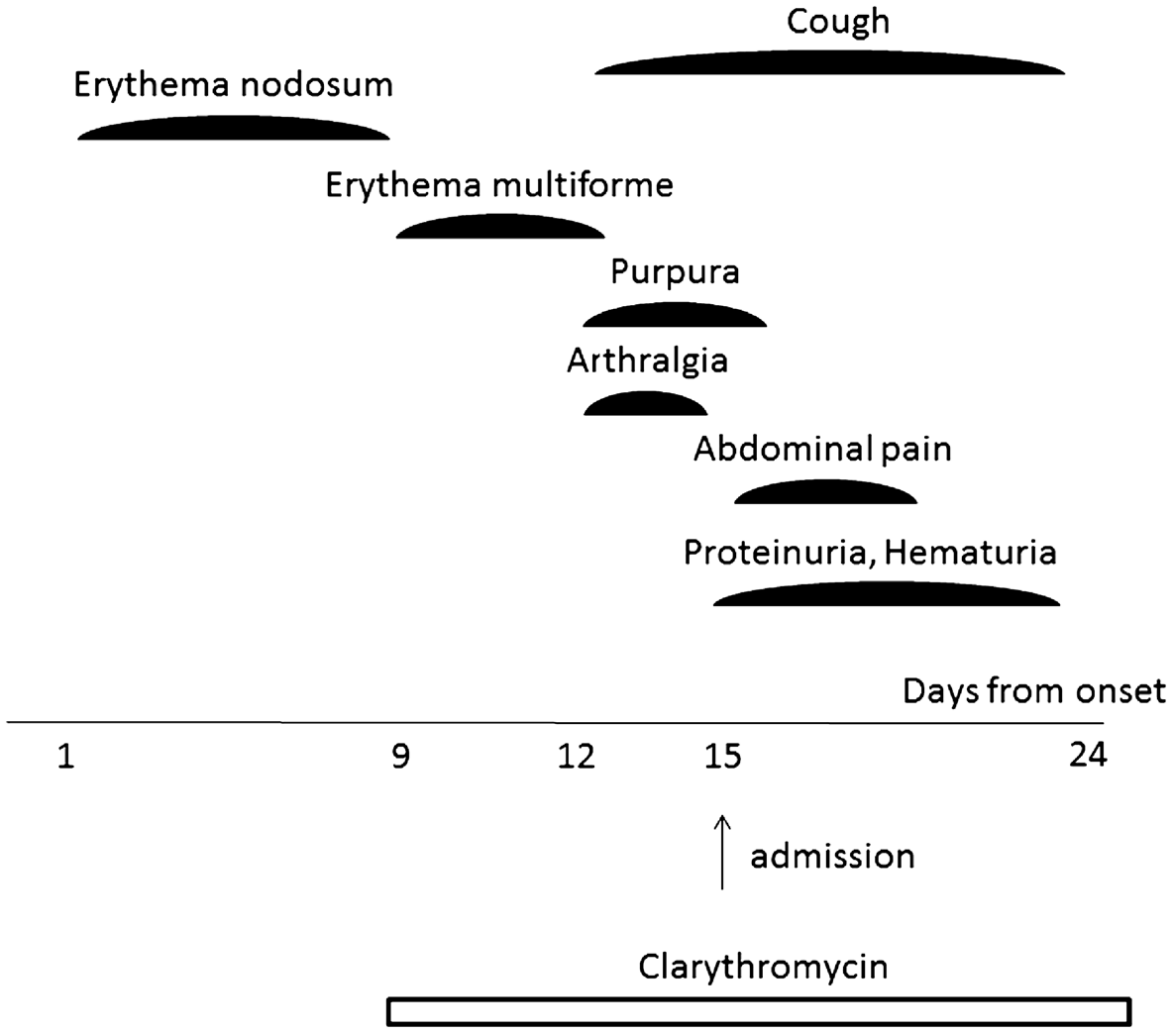
Clinical course.

**Figure 2:**
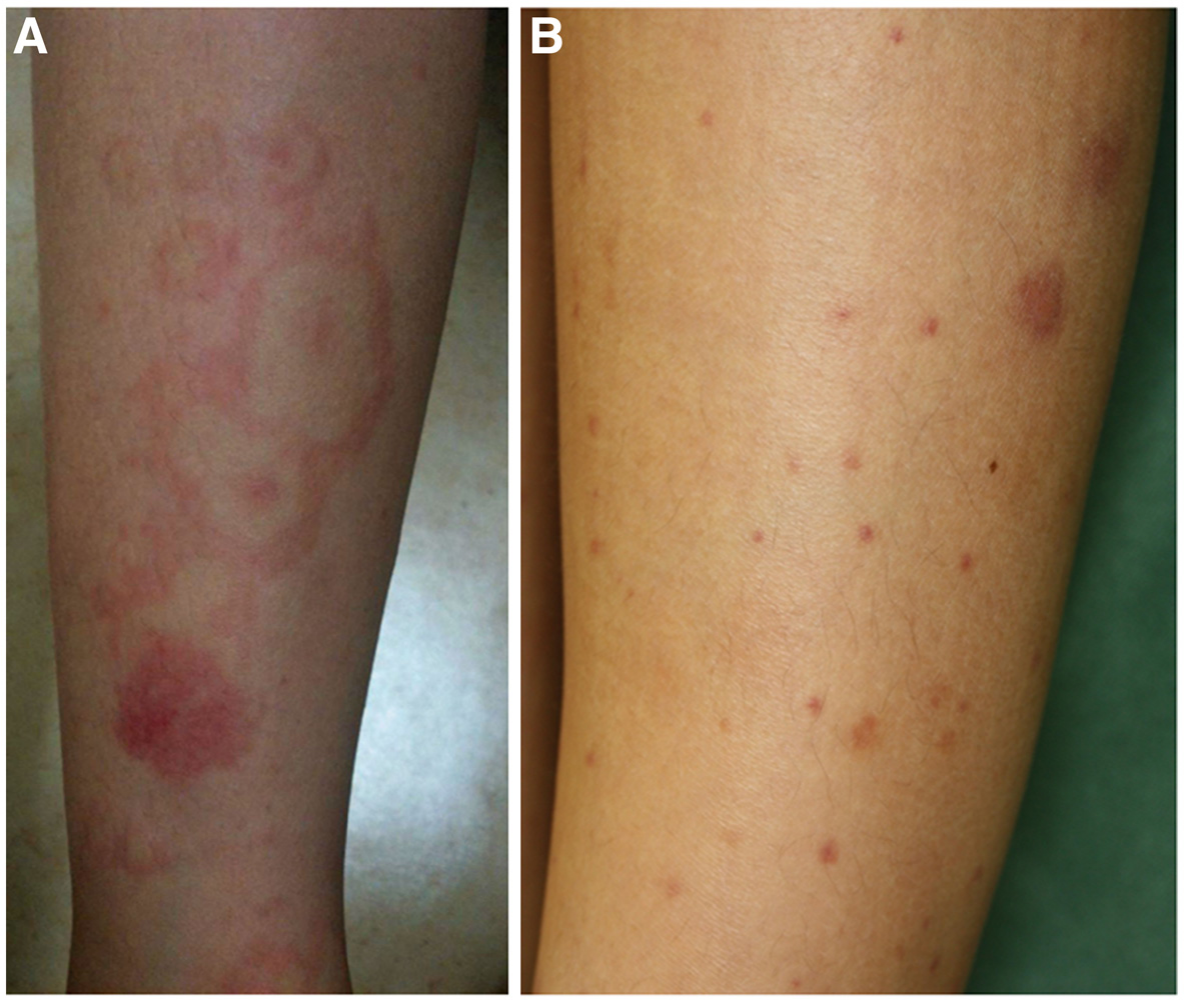
**The cutaneous manifestations of Henoch-Schönlein purpura in our patient.** (**A**) Erythema multiforme. Multiple papular, macular and target lesions of the right lower leg 6 days before admission. (**B**) Henoch-Schönlein purpura. Purpuric rash over the right lower leg was observed on admission.

A previously healthy 8-year-old Japanese girl was referred to our hospital with a 2-day history of symmetrical, multiple, round, light pink, tender erythematous nodules on her lower legs and arthralgia of her ankles 13 days before admission (Figure 1). Their sizes were approximately 2cm. The diagnosis of erythema nodosum was made. The lesions gradually improved and disappeared 7 days before admission; however, the patient then developed multiple maculopapular and targetoid lesions over her lower legs 6 days before admission (Figure 2A). No involvement of the mucous membranes was found. The diagnosis of erythema multiforme was made. The physical examination also revealed tonsillitis. Specific serum immunoglobulin M (IgM) antibodies for *M. pneumoniae* were positive, and clarithromycin therapy (300mg/day) was started. The lesions gradually improved and disappeared 3 days before admission (Figure 1). The patient had a mild cough; arthralgia of both hands; painful, localized edema on her scalp bilaterally; and a purpuric rash over her hip and lower legs concurrently 3 days before admission (Figure 2B). She also had abdominal pain and was admitted to our hospital. She was born in Japan and had not traveled out of Japan. She had no animal or insect bites. On admission, her body temperature was 36.8°C, heart rate 106 beats/minute and blood pressure 96/50mmHg. She had no weight loss. She had a purpuric rash over her hip and lower legs. The remainder of the examination was normal. Laboratory examinations showed a slightly elevated C-reactive protein level of 0.5mg/dl (normal <0.4mg/dl), white blood cell count of 8.44 × 10^9^/L, hemoglobin of 13.0g/dl; platelet count of 220 × 10^9^/L, normal liver and kidney function, and urinalysis with 2+ protein and 1+ blood. Complement studies showed normal C3 of 119mg/dl (normal, 86mg/dl to 160mg/dl), C4 of 22mg/dl (normal, 17mg/dl to 45mg/dl) and slightly elevated CH50 of 59U/ml (normal, 30U/ml to 45U/ml). A culture from the throat revealed normal flora only. The test for anti-streptolysin O antibody was negative. Hepatitis B surface antigen and hepatitis C antibody were negative. The titer of complement fixation tests for *M. pneumoniae* was 1:20480. Chest radiograph revealed no significant lung abnormalities. On the basis of the appearance of the latest rash and the associated symptoms, the diagnosis of Henoch-Schönlein purpura was made. The patient was treated with clarithromycin (300mg/day) for 3 weeks. Her symptoms improved gradually and disappeared 2 weeks later.

## Discussion

A wide variety of cutaneous manifestations have been described (Table 1). The most common manifestation of *M. pneumoniae* infection is the exanthematous or maculopapular eruption. This reaction occurs in of 8% to 33% of cases [1]. Erythema nodosum, urticaria and Stevens-Johnson syndrome are noted most often. The remaining cutaneous manifestations are rare [2].

**Table 1:** **Skin manifestations associated with *Mycoplasma pneumoniae *** **infection**

**Skin manifestation**	**Frequency**
Common	
Exanthematous skin eruptions	8% to 33%
Erythema nodosum	8%
Urticaria	7%
Stevens-Johnson syndrome	1% to 5%
Rare	
Bullous erythema multiforme	
Pityriasis rosea	
Henoch-Schönlein purpura	
Toxic epidermal necrolysis	
Kawasaki disease	
Subcorneal pustular dermatosis	
Thrombotic thrombocytopenic purpura	
Sweet’s syndrome	
Raynaud’s phenomenon	
Reiter syndrome	
Urticarial vasculitis	
Gianotti-Crosti syndrome	

The precise mechanisms by which *M. pneumoniae* produces a variety of cutaneous manifestations are poorly understood. Most cutaneous lesions are thought to be caused by the host response to antigens on these microbes rather than by the organisms themselves. Host immune responses such as immune complex-mediated injury, cytotoxic T-cell-mediated immune responses and autoimmune reactions have been speculated to play a crucial role in the development of cutaneous manifestations in patients with *M. pneumoniae* infection [3]. Patients with Henoch-Schönlein purpura likely have IgA-containing circulating immune complexes. Cytotoxic T cells have been implicated in patients with widespread vasculitis, such as in those with Kawasaki disease. Kano *et al*. [4] reported the cases of three patients with *M. pneumoniae* infection in a single family, each of whom manifested erythema nodosum, purpura and acute urticaria. They hypothesized that the variations in cutaneous manifestations of *M. pneumoniae* infections can be attributed to the immaturity of the adaptive immunity of a host. A feature of the present case is that different skin manifestations were observed in a single patient during the course of *M. pneumoniae* infection. This indicates that the type of cutaneous manifestation might not correlate with the maturity of the immune system in a given individual.

The most common cutaneous manifestations of *M. pneumoniae* infection, exanthematous or maculopapular eruption, may be either localized or confluent. These eruptions are self-limited, and no therapy for the cutaneous eruption is needed. Erythema nodosum is a reaction pattern caused by a variety of infections, including *M. pneumoniae*. In a previous report, it was shown that *M. pneumoniae* was the cause in 8% of patients with erythema nodosum [5]. Erythema multiforme is less common in *M. pneumoniae* infection. Thirty-five cases have been reported in the literature [6,7]. As shown in Figure 2A, patients with bullous erythema multiforme show target lesions, predominantly on the extremities, which spread to the trunk. Mucous membrane involvement occurs in most cases. Treatment includes topical corticosteroids for cutaneous lesions, local pain control with agents such as lidocaine solution, topical gel applied to painful oral lesions and treatment with appropriate antibiotics. *M. pneumoniae*-related Henoch-Schönlein purpura is a rare complication. A potential complication of *M. pneumoniae* infection and Henoch-Schönlein purpura is glomerulonephritis. Twenty-four cases of Henoch-Schönlein purpura-associated nephritis without skin findings have been related to *M. pneumoniae* infection [8]. Our patient is the first case with mild glomerulonephritis with skin lesions caused by *M. pneumoniae* infection. Fortunately, glomerulonephritis in our patient was mild. However, it is a serious internal complication of *M. pneumoniae* infection that should be screened for both during and after active infection. The underlying condition should be treated as appropriate with immunosuppressive agents, such as oral prednisone, if necessary.

It is still unclear whether drug ingestion contributes to skin reactions in *M. pneumoniae* infections. It has been reported that 17 (60%) of 29 *M. pneumoniae*-infected patients received antibiotics prior to the development of exanthema [9]. In a study comparing azithromycin with erythromycin or amoxicillin/clavulanate, eruption was seen in 6.1% of patients taking azithromycin/clavulanate compared with 12.3% in those receiving erythromycin. Of the total group examined, 29.5% had *M. pneumoniae* infection [10]. It is possible that the combination of *M. pneumoniae* infection and drug exposure could increase sensitivity to the development of drug exanthemas.

The case of our patient emphasizes the importance of considering *M. pneumoniae* infection in the differential diagnosis of a patient with a wide variety of skin manifestations. *M. pneumoniae* infection is frequently considered in the differential diagnosis of patients with respiratory illnesses. This contrasts with patients who present with non-respiratory symptoms in the context of a recent or current unrecognized *M. pneumoniae* infection, for whom this pathogen is rarely considered in the initial differential diagnosis. An awareness of the varied patterns of cutaneous disease is essential for the early diagnosis and treatment of patients with manifestations of *M. pneumoniae* infection. Skin manifestations could appear even in cases without pneumonia, such as in our patient. A chest radiograph alone is not sufficient to make the diagnosis. Serological examinations are recommended.

## Conclusion

We report the case of a patient with sequentially appearing skin manifestations, including erythema nodosum, erythema multiforme and Henoch-Schönlein purpura associated with *M. pneumoniae* infection. An awareness of the varied patterns of cutaneous disease is essential for the early diagnosis and treatment of patients with manifestations of *M. pneumoniae* infection.

## Consent

Written informed consent was obtained from the patient’s legal parent for publication of this manuscript and any accompanying images. A copy of the written consent is available for review by the Editor-in-Chief of this journal.

## Competing interests

The authors declare that they have no competing interests.

## Authors’ contributions

MS wrote the manuscript and was responsible for literature research. YH, TM, YS and AY were involved in critically revising the manuscript for important intellectual content. All authors read and approved the final manuscript.
